# Non-Mulberry and Mulberry Silk Protein Sericins as Potential Media Supplement for Animal Cell Culture

**DOI:** 10.1155/2016/7461041

**Published:** 2016-07-19

**Authors:** Neety Sahu, Shilpa Pal, Sunaina Sapru, Joydip Kundu, Sarmistha Talukdar, N. Ibotambi Singh, Juming Yao, Subhas C. Kundu

**Affiliations:** ^1^Department of Biotechnology, Indian Institute of Technology Kharagpur, West Bengal 721302, India; ^2^Central Muga Eri Research and Training Institute, Lahdoigarh, Jorhat, Assam 785700, India; ^3^The Key Laboratory of Advanced Textile Materials and Manufacturing Technology of Ministry of Education, College of Materials and Textiles, Zhejiang Sci-Tech University, Hangzhou 310018, China

## Abstract

Silk protein sericins, in the recent years, find application in cosmetics and pharmaceuticals and as biomaterials. We investigate the potential of sericin, extracted from both mulberry* Bombyx mori* and different non-mulberry sources, namely, tropical tasar,* Antheraea mylitta*; muga,* Antheraea assama*; and eri,* Samia ricini,* as growth supplement in serum-free culture medium. Sericin supplemented media containing different concentrations of sericins from the different species are examined for attachment, growth, proliferation, and morphology of fibrosarcoma cells. The optimum sericin supplementation seems to vary with the source of sericins. The results indicate that all the sericins promote the growth of L929 cells in serum-free culture media; however,* S. ricini* sericin seems to promote better growth of cells amongst other non-mulberry sericins.

## 1. Introduction

Silk protein sericin (glue protein) is a water soluble glycoprotein that shields the fibroin fibres present in the cocoon. This cocoon structure protects the pupae from the different environmental conditions, natural calamities, and predators (particularly for non-mulberry silkworms) stress. The glue-like strong adhesive nature of this protein is attributed to the hydrogen bonding capability of the enormous number of hydroxyl amino acids present in it. The removal of sericin from the silk fibres is carried out by the different processes called degumming. The sericins are mostly discarded as waste products during silk fibre processing in the textile industries, which now find place in economics. If the sericins are recovered and recycled properly, then this may play significant role in social benefits. Sericin recently finds widespread applications in cosmetic industry, as antioxidant and antiapoptotic compound, as support for enzyme immobilization, as supplement in animal cell culture media, as dietary supplement, and also as biomaterial for cell culture, drug, and gene delivery [1–8]. Addition of 0.5% sericin to cell culture medium improved the resistance to oxidative stress and quality of bovine embryos* in vitro* [9].

Sericin is isolated from the silk cocoons by the degumming process, which takes the advantage of the solubility of sericin in boiling aqueous solutions containing reagents like soap, alkali, synthetic detergents, urea, organic acids, and proteolytic enzymes. The most common methods used for the removal of sericin from cocoons are either by heating or by alkali treatment [10]. Mosher and Rayon in 1934 isolated sericin by boiling cocoons in hot water and separated them as water soluble and water insoluble fractions [11]. Usually in the silk industry, degumming of cocoons is carried out by alkaline condition utilizing either Na_2_CO_3_ or NaOH followed by boiling for 30 mins. Three types of polypeptides of mulberry silk sericin are obtained using isolation buffer, which contains 8 M urea, 1% SDS, and 2%  *β*-mercaptoethanol for 30 mins at room temperature, followed by heating at 80°C [12]. For isolating sericin in the native condition, the cut cocoons and peduncle pieces are soaked in 1% NaCl solution at room temperature overnight with shaking at 100 rpm followed by precipitation and resolubilization [13–15]. Sericin displays the ability to self-assemble* via* multiple aggregation mechanisms [16].

Sericin obtained from mulberry* Bombyx mori* contains a group of proteins ranging from 20 to 400 kDa and has an unusually high serine content (40%) along with significant glycine content (16%) [6]. Secondary structure of sericin from the cocoons of* B. mori *reveals the presence of *β*-sheet structure along with random coils [17]. The sericin of Indian non-mulberry tropical silkworm* Antheraea mylitta* has three prominent polypeptides of 70 kDa, 200 kDa, and a higher fraction of more than 200 kDa [14].

Low molecular weight sericin is reported to have myriad applications in biomedical, cosmetic, and pharmaceutical industries, as bioconjugates in drug delivery and graft copolymers. Sericin from* B. mori* mutant silkworm, sericin hope, a mutant of* Bombyx mori* and deficient of fibroin, is shown to be a new natural silk biomaterial for dermal equivalent for grafting. The fibroblast-keratinocyte coculture on 3D sericin hope matrix model is reported to be an alternative to* in vitro* skin replacement grafts [7].* A. mylitta* sericin coated titanium surfaces are also reported to have potential application in titanium based medical implants [18]. Mulberry sericin is used as blended biomaterial scaffolds to promote healing in injured tissues [19, 20]. Non-mulberry sericin is also used to promote dermal reconstruction [21]. Therefore, the idea that the sericin may have a role in promoting growth and proliferation of cells apart from its uses as a biomaterial holds promise. Sericin from mulberry* B. mori *is shown to accelerate cell proliferation in serum-free mammalian cell culture [22]. Apart from its applications in tissue engineering and healthcare industries, sericin is also reported to act as an alternative to serum in the culture of islet cells [23, 24]. Sericin is shown to prevent cell death and promote cellular growth in Sf9 insect cells after acute serum deprivation [25]. A novel serum-free freezing medium consisting of PBS, 1% (v/w) sericin, 0.5% (v/w) maltose, 0.3% (v/w) proline, 0.3% (v/w) glutamine, and 10% DMSO is developed and is found to be better than the conventional serum containing freezing media for the cryopreservation of P3U1 myeloma cell line, Chinese-hamster ovary cells, human dermal fibroblasts, human epidermal keratinocytes, the rat pheochromocytoma cell line PC12, and insect cell line Sf9 [26]. Bovine embryos are preserved well in sericin supplemented serum-free freezing media [27]. Serum-free medium containing sericin is shown to be suitable not only for cell culture but also for cryopreservation rat islets [28]. It has been shown that sericin may substitute for FBS in the freezing medium for primary hMSCs but cannot substitute for DMSO [29]. Though serum is widely used as a growth supplement in cell culture media, some potential disadvantages of serum limit its use in pharmaceuticals industries. These are (1) higher level of contaminants, the protein concentration in 10% serum being 6,200–10,1000 mg/L; (2) presence of various other components besides growth factors; (3) potential source of infectious agents, viral, bacterial, and fungal contamination of serum; and (4) high cost and availability [30]. The role of sericin in the growth of animal cells holds an advantage over serum. This holds true in cases where high level of purity of recovered cellular products is desired [22]. Moreover, silk sericin is an inexpensive alternative to serum in this regard. The sericins are low-cost abundant waste/by-product of silk textile industries and until now are underutilised. The application of sericin protein as biomaterial is documented in the literature [1, 6]. The useful properties of sericin like being antioxidant and antiapoptotic, hydrophilicity, and potential to promote cell attachment and growth provide the added characteristics for the use of sericin in the fabrication of various matrices (films, mats, 3D scaffolds, hydrogels, and nanoparticles) in diversified fields of tissue engineering and regenerative medicine (6). High solubility and weak structural composition make silk sericin very fragile and unsuitable for fabricating biomedical materials [31]. However, silk sericins are used to fabricate tenable materials for use in tissue engineering by some biochemical modifications [4, 31]. Our and other similar investigations [23, 24] indicate the ability of silk sericin to promote cell proliferation in serum-free culture media. This makes sericin an attractive component in the fabrication of biodegradable materials like hydrogels, films, and scaffolds, especially for the culture of cells that are susceptible to serum.

In this study, we investigate the biochemical, biophysical characteristics and the potential of the silk protein sericins as supplement to animal cell culture medium. The sericins are isolated from mulberry (*Bombyx mori*) and also different non-mulberry species (tropical tasar,* Antheraea mylitta; *muga*, Antheraea assama*; and eri,* Samia ricini*). The results indicate that the sericins may be used as potential supplements in place of serum for animal cell culture. This replacement with sericin may help future cell based tissue engineering and regenerative medicine practices.

## 2. Experimental

### 2.1. Materials

Live silk cocoons of mulberry silkworm,* Bombyx mori *(local farm), and non-mulberry Indian tropical tasar,* Antheraea mylitta *(our IIT farm), obtained from West Midnapore District, West Bengal State; non-mulberry muga silkworm* Antheraea assama*/*Antheraea assamensis* obtained from Cooch Behar District, West Bengal State; eri* Samia ricini/Philosamia ricini* silk cocoons obtained from Jalpaiguri Silk Farm, West Bengal State, India; and sericin hope (mutant of* B. mori*) obtained from China were collected for this study. The fine chemicals (St. Luisa, Sigma, USA, and Merck, India); protein molecular weight marker (Amersham, UK, Fermentas); Alamar Blue (Invitrogen, USA); cell culture grade chemicals, namely, Dulbecco's modified eagle medium (DMEM), fetal calf serum, trypsin-EDTA, and penicillin-streptomycin antibiotics (Gibco BRL, USA); and rhodamine-phalloidin and Hoechst, 33342 (Molecular Probes, USA), were purchased for this experimentation. Murine fibrosarcoma cell line, L929 (National Centre for Cell Science (NCCS), Pune, India), was maintained in DMEM supplemented with 10% fetal bovine serum (FBS) and 50 *μ*g/mL penicillin-streptomycin in 5% CO_2_ incubator till they attained confluency.

### 2.2. Isolation of Silk Sericins from the Silk Cocoons

The sericins were isolated individually from mulberry (*B. mori*) and non-mulberry (*A. mylitta, S. ricini, A. assama, *and sericin hope (containing about 98% sericin)) silk cocoons following the protocols modified from Zhang et al. [32] and Dash et al. [13]. In brief, the cocoons were cut into small pieces, degummed by boiling the cocoons in deionised water with a mass to liquid ratio (MLR) (w/v) of 1 : 20 under pressure using an autoclave at 110–120°C for 60 mins in cases of non-mulberry species (*A. mylitta, S. ricini, *and* A. assama*) and 30 mins for mulberry species (*B. mori *and sericin hope). Another extraction method as described by Takasu et al. [12] was followed for analysing the different polypeptide fractions of sericins present in the different species. The cocoon pieces were weighed and soaked in 8 M urea containing 2% *β*-mercaptoethanol and 1% SDS, followed by incubation at 80°C for 5 minutes. The sericin solutions were centrifuged at 800 rpm for 10 minutes and supernatant so obtained was dialyzed using cellulose tubes (3.5 kDa) against deionised water for 8–12 hrs with regular change of water intermittently. They were filtered using 0.45 *μ*m pore size filters. The sericin powders were obtained individually by lyophilizing the sericin solutions and stored at 4°C until use.

### 2.3. Scanning Electron Microscopy (SEM)

SEM images of the degummed cocoons were obtained after gold sputtering using a JEOL JSM-5800 scanning electron microscope with incident electron beam energy of 1 keV and a working distance of 6 mm.

### 2.4. Characterization of Sericins

#### 2.4.1. Estimation of Molecular Weight through SDS-PAGE

The molecular weight distribution of the sericin obtained using urea method [12] and the autoclave method was verified by SDS-PAGE. About 0.1–0.2 mg of the sericin samples that were isolated from the cocoons of* B. mori, A. mylitta, S. ricini, and A. assama *using hot boil degumming were incubated with Laemmli sample loading buffer. The proteins were loaded onto a 5% stacking gel cast on the top of an 8% SDS polyacrylamide gel (Merck) and electrophoresed. The gel was run at a constant voltage of 80 V for approximately 3 hrs so that the protein is resolved efficiently. After electrophoresis, the gel was stained with Coomassie Brilliant Blue R-250 (Sigma, USA) and destained in a methanol/water 1 : 1 solution that contained 20% acetic acid. To visualize the bands not observed by Coomassie stain, silver staining [33] was performed. Briefly, the gels were immersed in a fixative solution (40% methanol, 10% acetic acid, and 50% deionised water) for 1 hr. They were then washed with 30% ethanol thrice for 20 minutes each after which the gels were sensitized in the reductant (0.02% sodium thiosulphate) for 2 mins and washed thoroughly with deionised water. The gels were then stained with 0.1% silver nitrate (Merck) solution and 0.02% formaldehyde (Merck) for an hour, washed, and developed using 3% sodium carbonate (Sigma), 0.05% formaldehyde (Sigma, USA), and 0.5% sodium thiosulphate (Sigma, USA) until the bands appeared. When the desired intensity was achieved, the reaction was terminated using 5% acetic acid (Merck) solution.

#### 2.4.2. Circular Dichroism (CD) Spectroscopy

The circular dichroism (CD) studies of cocoon sericins from different species were performed on a JASCO J-810 spectropolarimeter using a 0.1 cm path length quartz cell at 20°C. The spectra were corrected for the baseline. The UV spectrum of the individual sericins was collected at a protein concentration of 0.1% (w/v) in water. The data points were recorded with a step resolution of 0.5 nm, time constant of 1 s, sensitivity of 10 m deg, scan speed of 50 nm/min, and spectral bandwidth of 2 nm. In order to reduce error and noise, each spectrum was an average of three scans over 400–190 nm. The background spectra were acquired from the same solvent. The spectra were corrected for the baseline and normalized to protein concentration in order to obtain the mean residue molar (deg cm^2^/d mol). The percentages of *α*-helix, *β*-sheet, turns, and random coil were determined using the standard protein secondary structure estimation program provided inbuilt with JASCO J-810 spectropolarimeter [34].

#### 2.4.3. Fourier Transform Infrared (FTIR) Spectroscopy

FTIR analysis of the sericin protein (powders) was carried out using an FTIR spectrometer (Thermo Nicolet Corporation NEXUS-870) with a resolution of 2 cm^−1^ with a scan range of 500 cm^−1^ to 2000 cm^−1^.

#### 2.4.4. Thermogravimetric Analysis

Thermogravimetric analysis (TGA) was run under the flow of nitrogen gas from 30 to 650°C at a scanning speed of 20°C/min using Pyris Diamond.

### 2.5. Cell Culture

Approximately 10^3^ mouse fibrosarcoma cells (L929) per well were seeded in 24-well tissue culture plates with DMEM (Dulbecco's Modified Eagle's Medium), supplemented with 10% fetal bovine serum, and allowed to get attached to the plate surface for 24 hrs. The cells were then starved for 24 hrs by supplying serum-free (incomplete) DMEM. Sericin supplemented medium was prepared initially by adding different concentrations of sericin solution to incomplete DMEM. Finally, media containing 2 different concentrations (0.05% and 0.1%) of sericin of each of the 4 species were prepared and used as supplement in cell culture medium. Each of these sericin supplemented media was filtered under sterile conditions using a 0.22 *μ*m filter. The previously starved cells were treated with the different concentrations of sericin supplemented media over a period of 3 days. L929 cells growing in serum supplemented DMEM and in serum-free DMEM were used as controls.

#### 2.5.1. Cell Viability

The cell viability was estimated by the reduction of Alamar Blue as substrate. The cells grown in sericin supplemented media were incubated with Alamar Blue at 37°C for 3 hrs. At the end of the assay, the supernatant was taken and the absorbance was measured at 570 nm and 600 nm. The percentage of Alamar Blue reduced was plotted as a function of cellular metabolic activity and viability. Alamar Blue assay was performed after 24 hrs and 72 hrs of sericin treatment of the cells.

#### 2.5.2. Cell Morphology

Morphology of the cells was studied by visualizing the cells under inverted phase contrast microscope and fluorescence microscope (Leica, Germany).

#### 2.5.3. Cell Attachment

L929 cell attachment in sericin supplemented DMEM was studied by measuring the number of cells attached to the surface of tissue culture well as a function of time. Confluent L929 cells grown in serum supplemented DMEM were trypsinized and 10^5^ cells per well were seeded onto the wells containing 0.05% sericin supplemented DMEM of* B. mori, A. mylitta*,* A. assama*, and* S. ricini.* The cell attachment study was performed after 2, 4, 6, 8, 10, and 12 hrs of seeding. Media supernatant was removed at every time point and the number of unattached cells was enumerated. The number of cells attached as obtained by deducting the number of unattached cells from total number of cells seeded was plotted against time. (We have not carried out any further work on sericin hope being a mutant cocoon, which contains mostly sericins. This unique bioengineered silk sericin hope needs to be evaluated more on different aspects).

### 2.6. Statistical Analysis

Experiments were run in triplicate per sample and each experiment was conducted at least thrice. All data were expressed as mean ± standard deviation (SD) for *n* = 3. A single-factor analysis of variance technique was used to determine the statistical significance of the results.

## 3. Results

### 3.1. Isolation of Sericin from the Cocoons

**Figure 1:**
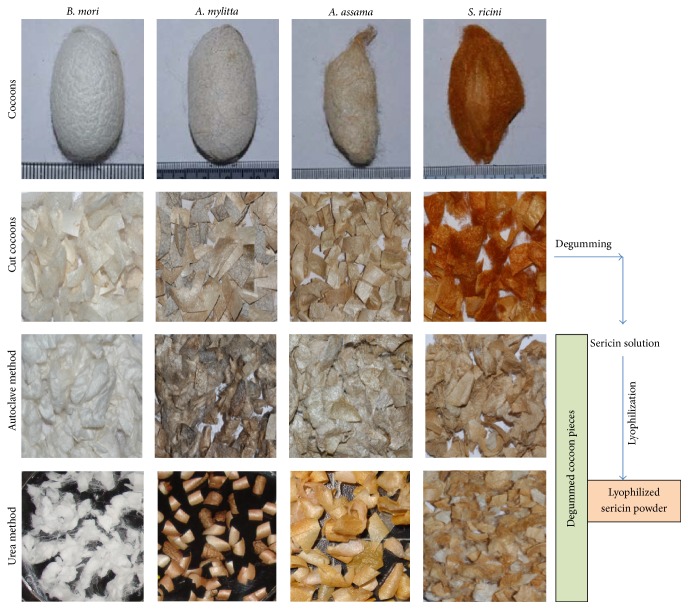
Degumming of silk cocoons of different species of silkworms using different methods of isolation of sericins.

Sericins are isolated from the cocoons of* A. mylitta*,* A. assama*,* S. ricini*, and* B. mori* (Figure 1). They are processed, that is, dialyzed to remove salts, fats, and others, and finally concentrated. Different extraction methods produce variable yields of extracted sericin depending on the physical properties of cocoons. Silk sericin can be extracted by degumming silk cocoons using various chemical agents like urea, sodium chloride, sodium carbonate, and sodium hydroxide. Highest sericin yield is reported to be achieved via sodium carbonate treatment while urea method produces lowest amount of sericin [10]. Additionally, different species of silk are also known to have variable yields of sericin for any extraction method [10]. Nonchemical (autoclave) method shows variable degumming ratio in mulberry (18–21%) and non-mulberry species (6–11%). At times, the variation in extracted amount of sericin from non-mulberry is very low and unpredictable even in the same species. What we understand is that several factors are involved in the variation of sericin yields like age/freshness, eco-races/strain, season, place of collection, type of crops (multivoltine/bivoltine), storage conditions, contamination (being wild), rearing procedure, handling of cocoons, and others [10]. The variations in the sericin content in mulberry and non-mulberry silk cocoons result in the differences of sericin yield in these species.

### 3.2. Scanning Electron Microscopy (SEM)

**Figure 2:**
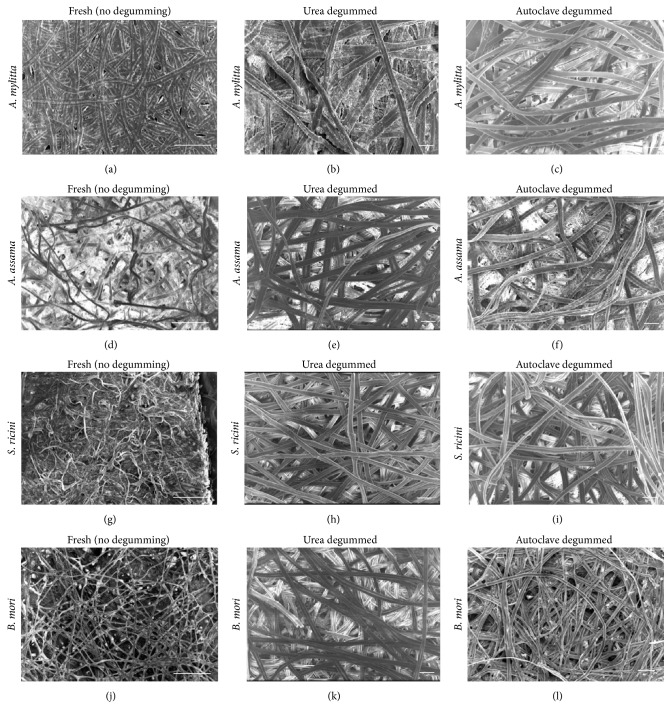
Scanning electron micrographs of the cocoon pieces of mulberry and non-mulberry silks. The cocoons are observed before (50x) and after degumming (100x) using urea and autoclave degumming methods. Scale bar represents 100 *μ*m.

SEM is carried out to observe the effect of degumming on the microstructures of the fibres of the cocoons.  Figure 2 shows the SEM micrographs of cocoon pieces before and after degumming by 8 M urea and boiling under pressure (autoclave method). The micrographs of nondegummed silk cocoons have white striations, indicating sericin, between the fibroin fibres, whereas the degummed fibres have minimal sericin between the fibres. The fibres are distinctly visible and well separated from one another due to the removal of the glue protein sericin, which holds them together.

### 3.3. Characterization of Silk Sericins

#### 3.3.1. Estimation of Molecular Weight through SDS-PAGE

**Figure 3:**
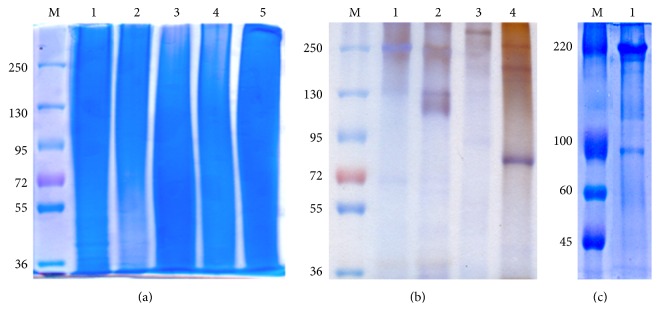
SDS-PAGE (8%) analysis of 0.1% sericin solutions from cocoons of* A. mylitta*,* B. mori*,* S. ricini*, and* A. assama*: (a) isolated by autoclave method: Lane 1: sericin hope, Lane 2:* B. mori,* Lane 3:* S. ricini*, Lane 4:* A. assama*, and Lane 5:* A. mylitta*; (b) isolated by urea method: Lane 1: sericin hope, Lane 2:* B. mori*, Lane 3:* S. ricini*, and Lane 4:* A. mylitta*. (c) Lane 1:* A. assama* isolated by urea method. The protein molecular weight standards are indicated by the numbers on the left. M: molecular weight marker.

The sericins of different silkworm species extracted by autoclave method appear as smears in the 8% gel after electrophoresis (Figure 3). Heating of the cocoons under high pressure and temperature degrades the protein. On the other hand, sericin isolated by urea method separates the protein into various fractions, which appear as bands in the gel after the run (Figure 3). Marked differences are seen in sericin extracted from mulberry species from that in non-mulberry species. The sericin fractions obtained in case of* B. mori* and* A. mylitta* are comparable to results obtained previously [14].* B. mori* sericin shows bands of 250 kDa and 130 kDa, some in the range of 120–130 kDa, and a lower fraction of 17 kDa [35].* A. mylitta* sericin comprised mainly three fractions of approximately 250 kDa, 200 kDa, and 70 kDa.* S. ricini* has two bands, one greater than 300 kDa and one in the range of 200–250 kDa (Figure 3). For* A. assama* one fraction greater than 250 kDa and another approximately 90 kDa are observed. As inferred by SDS-PAGE, sericin represents a family of proteins with a diverse distribution of molecular weights. Distinct fractions of sericin polypeptides are isolated in the urea method. It is shown that L929 cells grown in medium containing sericin extracted by urea method exhibited cytotoxicity [36]; therefore, this method is not used for preparing sericin supplemented medium in our study. However, to illustrate the different bands or fractions of the sericins from* A. mylitta*,* A. assama*,* S. ricini*, and* B. mori* in SDS-PAGE, sericins extracted by urea method are employed. The other methods used for extraction of sericins provide smears in gels [36]. As sericin hope consists of mostly sericin protein, it is therefore employed for comparison with sericin of* B. mori*,* A. mylitta*, and* S. ricini* by SDS-PAGE. No further work, apart from SDS-PAGE, is carried out with sericin hope for comparison. The autoclave method gives a smear in SDS-PAGE indicating that the protein polypeptides are broken into smaller fractions. This autoclave method is chosen for further investigations as it is free of any toxic ions.

#### 3.3.2. Circular Dichroism (CD) Spectroscopy

**Figure 4:**
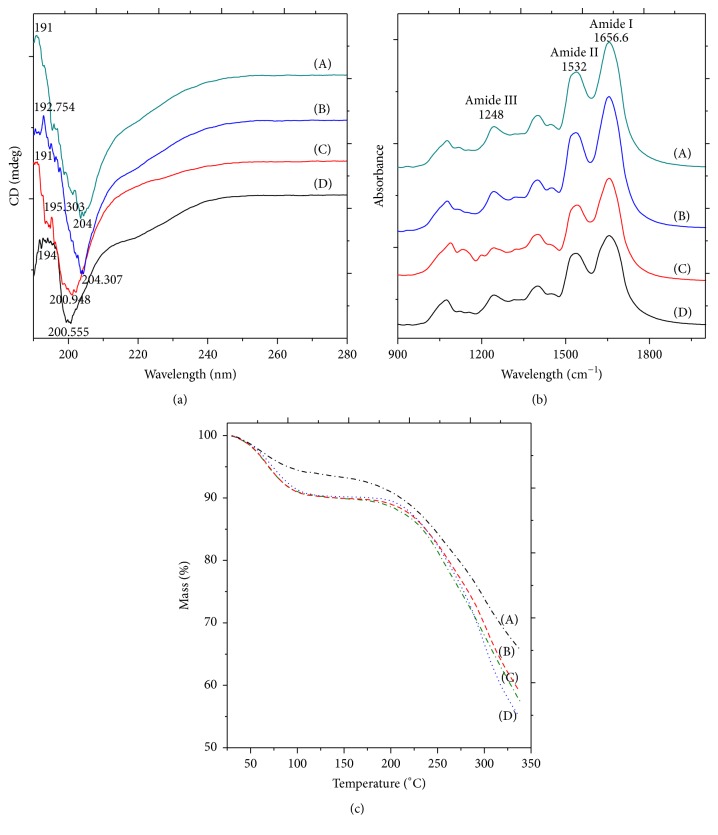
(a) CD spectra of 0.1 % (w/v) sericin solution from cocoons of different species: (A)* A. mylitta*, (B)* A. assama*, (C)* S. ricini*, and (D)* B. mori*. (b) FTIR spectrum of sericin powders from the various species: (A)* A. mylitta*, (B)* A. assama*, (C)* S. ricini*, and (D)* B. mori*. (c) TGA curves of lyophilized sericin powders of (A)* S. ricini*, (B)* A. mylitta*, (C)* A. assama*, and (D)* B. mori.*

The CD spectrum of the cocoon sericin solutions of all species shows one sharp negative band at around 200 nm assigned to random coil conformation (Figure 4(a)). A negative band at 218 nm reveals the presence of *β*-sheet. The alpha helix content is low because of the absence of double minimum at 222 nm and 208–210 nm and a positive band at 190 nm. The secondary structure data shows that the percentage of random coil is highest in* S. ricini*. Percentage of beta is highest in the wild species* A. assama*, followed by* A. mylitta *and* B. mori,* and the least in* S. ricini. *Small percentages of turns are seen in* S. ricini *and* B. mori*. They are absent in the other two species. Helix is present in very small amount in* A. mylitta *and negligible in* A. assama.* In spite of the presence of higher amount of glutamic acid and lower percentage of tyrosine, CD spectroscopy analysis of the secondary structure of the* A. mylitta* sericin reveals its *β*-sheet structure in native form. The *β*-sheet structure may be due to the polar zipper interaction through hydrogen bonding among abundant polar amino acids in the serine-rich repetitive motif [37]. It was reported previously that the *β*-sheet conformational structure of sericin in aqueous solution is stabilized by the hydration and electrostatic interactions [38].

#### 3.3.3. Fourier Transform Infrared (FTIR) Spectroscopy

All the species have similar peaks at 1656.58 cm^−1^ (Amide I), 1540–1543 cm^−1^ (Amide II), and 1242–1246 (Amide III) (Figure 4(b)). The peak at 1656.58 cm^−1^ suggests the presence of *α*-helix. The *β*-sheet aggregates help in stabilizing sericin in water, hence contributing to the strength of the cocoon and suitability as a biomaterial. The CD spectrum of all the species shows the presence of random coil conformation and *β*-sheets with a low content of alpha helix. In aqueous form, the transition from random coil to *β*-sheet is quicker whereas anhydrous form has fewer tendencies to change its conformation. Hence, in the FTIR, we find the protein of the lyophilized powder as random coil and alpha helix, whereas the transition from the random coil to *β*-sheet is observed in CD results of aqueous solution of sericin.

#### 3.3.4. Thermogravimetric Analysis

The thermogravimetric curves of sericin powders are shown in Figure 4(c). When comparing the graphs for the four samples, it is observed that the peaks in the DTG curve are the smallest for* S. ricini* sample and the largest for* B. mori*. This gives an indication that the rate of change in weight of the sample with time (or temperature) for* S. ricini* is more gradual than that for the sample of* B. mori*. Or in other words,* S. ricini* is thermally more stable than* B. mori.* A lower peak in the DTG curve means that the sample loses weight more steadily. Hence, the corresponding TGA curve is smoother and has lesser slopes at most times (and temperatures). By similar arguments and by observing the plots, the samples can be arranged in the following order of decreasing stability:* S. ricini, A. assama, A. mylitta, *and, lastly, sericin of* B. mori*.* S. ricini* is thermally more stable as compared to that of other species.

### 3.4. Cell Culture

#### 3.4.1. Cell Attachment

**Figure 5:**
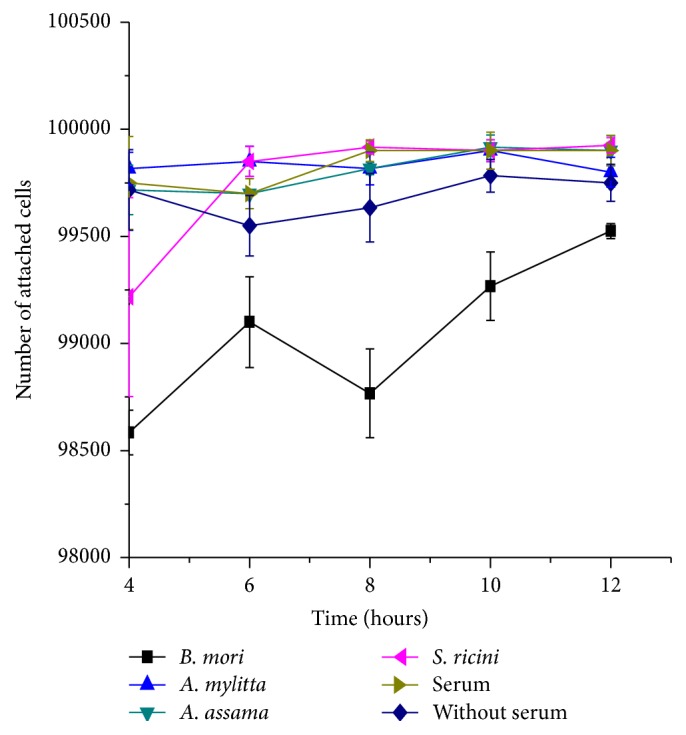
Time-dependent attachment of L929 cells growing in DMEM supplemented with 0.05% sericin of* B. mori, A. mylitta*,* A. assama*, and* S. ricini *sericin. Cells grown in DMEM supplemented with 10% serum and without serum were used as controls (error bars denote standard deviation for *n* = 3).

The key in the attachment result is the time taken by cells to reach maximum attachment of cells in DMEM supplemented with 0.05% sericin of* A. mylitta*,* A. assama,* and* S. ricini* is comparable to that in serum supplemented and serum-free DMEM, which show maximum cell attachment at 10 hrs after seeding (Figure 5). For media supplemented with 0.05% sericin of* B. mori*, maximum cell attachment is observed at 12 hrs after seeding, which is in accordance with similar attachment study reported earlier [39]. After 4 hours of cell seeding, the percentage of cells attached in the case of serum-free medium was 99.75% and it remained approximately the same after 12 hours of seeding. However, in case of the cells growing in* B. mori* sericin supplemented medium, 98.5% cells were attached at 4 hours while the number increased to 99.48% after 12 hours. Percentage of the cells attached in non-mulberry sericin supplemented media at 4 hours and 12 hours was similar to that in serum-free media. The attachment of cells in* B. mori* sericin supplemented medium was almost similar in comparison to non-mulberry sericin and serum-free media. We can also say that non-mulberry sericin supported attachment faster than mulberry sericin. The final number of cells attached at 12th hour for cells in serum-free medium and in mulberry supplemented medium does not have a statistical difference (*p*  value > 0.05). So, it can be concluded that somehow the mulberry sericin supplemented medium does promote cell attachment but at a rate slower than serum-free and non-mulberry sericin supplemented media. Furthermore, Tsubouchi et al. [40] reported that attachment is enhanced in mulberry sericin due to a repetitive fraction called sericin M (170 kDa). No sericin protein fraction other than the M fraction contributed to cell attachment. This 170 KDa protein fraction is absent in our SDS-PAGE results. This could be a reason for slower attachment of cells in mulberry supplemented medium.

#### 3.4.2. Cell Morphology

**Figure 6:**
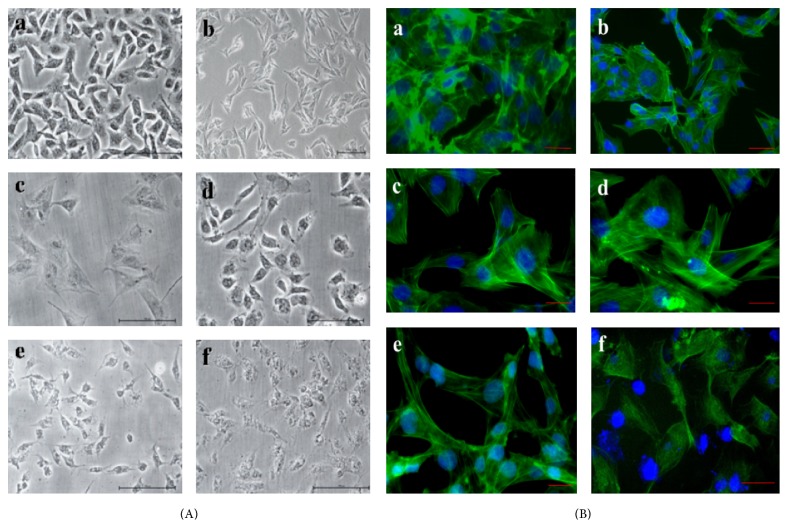
(A) Phase contrast and (B) fluorescence microscopic observations showing L929 fibroblasts growth and attachment on (a) 10% serum supplemented DMEM and (b) serum-free DMEM as controls and in DMEM supplemented with 0.05% silk protein sericins of* B. mori* (c),* A. mylitta* (d),* A. assama* (e), and* S. ricini* (f). Scale bar represents 10 *μ*m.

Cell morphology is studied by viewing the cells under inverted phase contrast microscope (Leica, Germany). The cells grown in normal complete DMEM are used as a control. In comparison with the control, the cells grown in sericin supplemented media show a marked morphology change as the concentration of sericin in the media is increased. The morphology of cells, as compared by phase contrast microscope, cultured in sericin supplemented media of* B. mori* and* A. mylitta* is comparable to the cells growing in 10% FBS supplemented control. However, morphology of the cells grown in* S. ricini* and* A. assama* sericin supplemented medium appears to be different (Figure 6(A)). Some morphological changes are observed when cells are cultured with* S. ricini* and* A. assama* sericin supplemented media. However, we have not studied the loss of phenotype or differentiation of cells into other types. The cells growing in control medium are typically spindle shaped. However, the sericin supplemented media did not negatively affect the actin cytoskeleton of the cells; fluorescence images show well-defined nuclei and actin microfilaments comparable to those of the cells grown in serum supplemented media (Figure 6(B)).

#### 3.4.3. Cell Proliferation

**Figure 7:**
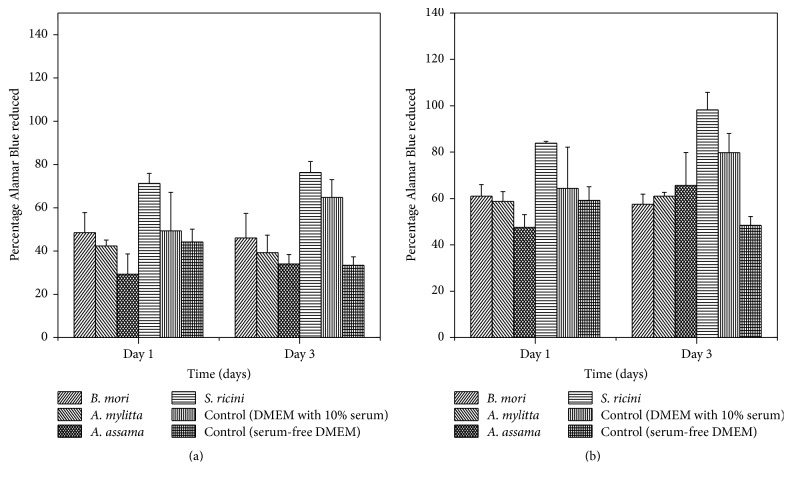
Estimation of cell viability and proliferation by Alamar Blue assay on L929 cells grown on 0.1% (a) and 0.05% (b) concentrations of sericin of* B. mori*,* A. mylitta*,* A. assama, *and* S. ricini, *10% FBS supplemented DMEM and FBS free DMEM (error bars denote standard deviation for *n* = 3).

Alamar Blue, a water soluble dye, is reduced by the cells growing in the medium, thereby changing the colour of the media from blue to pink which is measured colourimetrically. The number of viable cells correlates with the magnitude of dye reduction and is expressed as percentage of Alamar Blue reduced [41, 42]. Alamar assay conducted on L929 cells grown in sericin supplemented DMEM over a period of 3 days (Figure 7) shows that the cells not only are viable but are also proliferating. This is comparable to that of the cells grown in serum-free DMEM. While sericin of* S. ricini *helps to proliferate the cells better over a period of 3 days, the other non-mulberry and mulberry sericins do not show a marked difference in proliferation. Even the growth of cells in 0.1%* S. ricini* sericin supplemented medium is also significantly greater than the cells growing in 10% serum supplemented medium. This is also found to be true for 0.05%* S. ricini* sericin supplemented medium. Furthermore, the growth of cells in 0.05%* B. mori*,* A. mylitta*, and* A. assama* is found to be better than that of the cells growing in serum-free medium. There is a reduction on the growth of cells in serum-free medium from day one to day three. The cells grown in sericin supplemented media do not reflect the same. Based on the present cell culture assays the sericins of both mulberry and non-mulberry species appear to be beneficial to the growth of cells.

## 4. Discussion

Different sources of sericin show varied bands of different molecular weights depending on the silkworm species used as the source of the protein when extracted using urea. This may lead to different chemical and biological properties, while harsh treatments (high temperature and pressure) tend to degrade the protein leading to the appearance of a smear in the gel. Amongst them the common one is seen around 200–250 kDa along with certain low molecular weight sericin. Sericin-S, a small sericin having molecular weight 5 to 100 kDa of* B. mori*, is believed to act as a mitogenic factor in serum-free media. This accelerates the proliferation of hybridoma cells and T-lymphocyte cells [22]. Novel sericin-GIT medium, devoid of mammalian factors but containing sericin and other nutrients, helps in the proliferation of various cell lines, namely, HepG2, HeLa, SIRC, and L929 [43]. In our study, the silk fibroin fibres are seen to be clear of any remains of sericin when degummed properly indicating efficient extraction of sericin from the silk cocoon. Sericins show random coils and *β*-sheets in their secondary structures. This may be attributed to the polar interactions among the amino acids in the serine-rich motifs by hydrogen bonding leading to zipper like arrangements [37]. The transition from random coil to *β*-sheets in solution is stabilized by electrostatic interactions [38]. Thermal stability also differs in sericins. Sericin of* S. ricini* is seen to be most thermally stable amongst all. Sericins of mulberry and non-mulberry species are reported to have different biochemical properties [6]. While* B. mori* sericin comprises polypeptides ranging from 24 to 400 kDa, non-mulberry sericins also differ in molecular weights from one another. One major polypeptide fraction of 66 kDa is identified in sericins of* A. assama* and* S. ricini*, while* A. mylitta* sericin consists of several polypeptide fractions ranging from 30 to 200 kDa [6]. The two ranges of molecular weight-large sericin chain (MW 191–339 kDa) and small-size sericin (MW 61–132 kDa) are investigated for the apoptosis and proliferation of the colon cancer cells [44]. The smaller sericin had higher antiproliferative effects than that of the large sericin but neither of the sericin types (small or large sericin) affects the viability of the cells. So we can assume that the cell attachment will be similar, irrespective of the molecular weight of the sericins. Amino acid composition and secondary structure of* A. mylitta* sericin also vary from those of* B. mori* sericin [13]. Thus, the diversity of biochemical properties of silk sericins among different species of silkworms confers distinct biophysical properties to the sericins. The 400 kDa sericin found in the middle portion of the* B. mori* silk gland, particularly its 170 kDa fraction consisting of serine-rich repetitive domain, is reported to promote skin fibroblast attachment and activity. The sericins of anterior and posterior portions of* B. mori* silk grand show no biological effects in skin fibroblasts even though the posterior sericin has a 170 kDa fraction but no serine-rich amino acid composition [40]. Thus, attachment and proliferation of cells depend not only on the presence of specific polypeptide fractions but also on the amino acid content in the particular fraction. The difference in attachment of cells grown in sericin supplemented medium of different silkworm species in our study may be attributed to the differences in biophysical properties of the sericins.

Sericin has several beneficial properties and has already proved to be one of the natural biomaterials for different biomedical applications [6, 45, 46]. This work indicates that sericin can be used as media supplements. Investigations in the field of tissue engineering and regenerative medicine require growth supplements (growth factors or proteins), which are commonly provided by serum. This work indicates that sericin can support and enhance the cell growth equivalent to serum minimizing the limitation associated with the use of serum.

Replacement of the fetal bovine serum with mulberry* B. mori* sericin is reported to be successful in the culture of rat islet cells [23]. It is known that despite the benefits of serum in cell culture, it hinders the recovery of cellular product from media during downstream processing. The incorporation of sericin in the design of better serum-free media, therefore, may find better place in industries where cellular products of high purity are required. Media used for tissue culture may have significant effects on the growth and morphology of cells [47, 48]. In a departure from similar studies that report the use of* B. mori* sericin as a serum substitute in the growth of fibroblast cells [36, 49], our study shows that the low concentrations of both mulberry and non-mulberry sericin supplemented media can be used as supplements to serum-free media. The differences in the morphology of the cells are observed in cells cultured in media supplemented with sericin of* S. ricini* and* A. assama*. Sericin is known to affect different pathways due to its antioxidant properties [3, 50–52]. The morphological changes may be attributed to the effect of sericin on certain signalling pathway. Sericin supplemented media do not seem to negatively affect the actin cytoskeleton of the cells as fluorescence images show well-defined nuclei and actin microfilaments comparable to those of serum supplemented media (Figure 6(B)).

Sericins extracted by different methods were reported to exhibit different physical and biological properties [36]. Sericins consist of different fractions in each species. The extracted quantity of sericins and its fractions depend upon the source of materials, age, storage condition, and extraction protocol [10]. In the autoclave method, we observe only smears in the gels due to extreme conditions used in the extraction procedure. However, the individual fraction can be purified by other extraction methods but the detailed study based on the above parameters is yet to be carried out for each species. The sericins used in the cell culture of this study comprise all the fractions together of a particular species. Out of various methods of extraction, heat-degraded extraction of sericin proved to be least toxic and produced highest collagen in fibroblast cells and a concentration of 100 *μ*g/mL seemed to be optimal for use in serum-free growth medium [51]. Therefore, in our study, heat-extracted sericins were used in serum-free culture medium. The cells grown in sericin supplemented media from* A. mylitta* and* B. mori* depict not only good cell viability but also proper cell morphology that is comparable to serum supplemented media (Figures 6(A)(c, d) and 6(B)(c, d)). Therefore, as low as 0.05% sericins from* B. mori* and* A. mylitta *can be used as potential medium supplements for the culture of L929 fibroblast cells. The authors do not claim at this stage that sericin can completely replace serum supplementation in different kinds of cells. However, in dealing with delicate cell cultures that are prone to serum shock, the sericin may act as a growth supplement. The effects of concentration of sericin in the growth medium are reported to affect the cell viability and growth. In a similar study, mulberry sericin extracted by heat-degradation or autoclave method had been shown to enhance the proliferation of L929 cells in the medium supplemented with 0.03%* B. mori *sericin while the cell viability decreased considerably at higher sericin concentration of 0.3% [53]. Furthermore, Aramwit et al. reported that a low concentration of 8 *μ*g/mL heat-degraded sericin showed highest cell viability in mouse fibroblast cells while higher concentrations of sericin in the medium significantly decreased cell viability [36]. In our study, viability increased when the cells were grown in 0.05% sericin of non-mulberry species during the course of 3 days.* S. ricini* sericin showed the highest increase in viability. However, the viability of cells growing in 0.1% sericin did not change as effectively when compared with cells growing in 0.05% sericin supplemented medium. Thus, the results indicate that the growth and viability of cells depend on the concentration of sericin in the medium. The results indicate that further investigations are to be conducted to determine how sericin regulates the different cellular morphology and functions. This requires optimizing the dosage for each species of sericin for different cell lines. The present work attempts to present that both mulberry and different non-mulberry silk protein sericins play an important role in cell culture.

## 5. Conclusion

Sericins from mulberry and different non-mulberry species usually depict that more stable *β*-sheets conformation is solution, which adds to its integrity in aqueous media. Different molecular weights of sericin may be determining the difference in the properties of sericin from different sources like thermal stability and cytocompatibility. The low concentrations (0.05%) of all the different sources of sericins supplemented media show better cell growth than serum-free media. This indicates that sericin supports both the attachment and growth of the cells. The morphology of cells cultured in sericin supplemented media from mulberry* Bombyx mori* and non-mulberry* Antheraea mylitta* is comparable to that of the cells grown in medium supplemented with 10% FBS. There is a change in the morphology of the cells grown in* Samia ricini* and* Antheraea assama *sericin supplemented media and the cause for the change needs attention. Sericins of both mulberry and non-mulberry species have the potential to be used as a substitute for serum in media for the growth of cells. The sericins are low-cost, abundant, waste/by-product of textile industries and are underutilised materials. Further investigations are needed to understand how sericins aid in the growth and proliferation of cells and their behavior of different sources.
